# Anticancer Activity of the Antimicrobial Myristoylated Peptide Myr-B in HeLa Cells: Cytotoxic, Membrane-Disruptive and Proteomic Insights

**DOI:** 10.3390/ijms27093918

**Published:** 2026-04-28

**Authors:** Michele Costanzo, Francesco Maiurano, Marianna Caterino, Anna Rita Taddei, Sabrina Bianco, Simona Picchietti, Francesco Buonocore, Esther Imperlini

**Affiliations:** 1 Department of Molecular Medicine and Medical Biotechnology, University of Naples Federico II, 80131 Naples, Italy; michele.costanzo@unina.it (M.C.); marianna.caterino@unina.it (M.C.); 2 CEINGE—Biotecnologie Avanzate Franco Salvatore, 80145 Naples, Italy; biancos@ceinge.unina.it; 3 Department for Innovation in Biological, Agrofood and Forest Systems, University of Tuscia, 01100 Viterbo, Italy; francesco.maiurano@unitus.it (F.M.); picchietti@unitus.it (S.P.); 4 Center of Large Equipments, Section of Electron Microscopy, University of Tuscia, 01100 Viterbo, Italy; artaddei@unitus.it; 5 Department of Experimental and Clinical Medicine, University of Catanzaro Magna Græcia, Viale Europa, 88100 Catanzaro, Italy

**Keywords:** antimicrobial peptide, anticancer activity, N-myristoylated peptide, label-free proteomics, bioinformatics

## Abstract

Antimicrobial peptides (AMPs) are natural bioactive peptides produced by all organisms—from plants to insects, microbes and animals—and constitute a first line of defense. As they exhibit a broad spectrum of activity (antibacterial, antiviral, antifungal, antiparasitic, anticancer), strong efforts are being made to integrate AMPs into clinical use. AMPs are also being investigated as anticancer agents to overcome the side effects and/or resistance associated with current chemotherapies. In this context, we identified the natural AMP chionodracine from a new biological source: an Antarctic fish. Starting from the fragmentation of a chionodracine mutant peptide, a rational modular design approach was applied to develop three very short peptides (Pep-A, Pep-B and Pep-C), which were further modified with an N-terminal myristic acid lipid tail. The anticancer activity of the three N-myristoylated short peptides (Myr-A, Myr-B and Myr-C) was explored against the human cervical cancer HeLa cell line. The rationale behind this study is based on the previously reported antifungal activity of these myr peptides and on their ability to interact selectively with biological membrane-mimicking synthetic phospholipids without being particularly hemolytic or cytotoxic towards normal cells. We first demonstrated that myr peptides had cytotoxic activity against HeLa cells (IC_50_ from 32 to 47 μM) but spared healthy primary human fibroblasts, whereas the corresponding non-myr peptides failed to kill cancer cells. The peptide with no hemolytic activity and a low IC_50_, labeled Myr-B, was selected for subsequent analyses. Lactate dehydrogenase (LDH) assay and scanning electron microscopy (SEM) analysis revealed membrane damage and predominantly necrotic cell death in HeLa cells exposed to IC_50_ doses of the Myr-B peptide, compared with cells treated with Pep-B. To thoroughly investigate the molecular effects of Myr-B in HeLa cells, we employed high-resolution label-free shotgun quantitative proteomics coupled with bioinformatics. Our results showed that exposing HeLa cells to Myr-B led to the under-expression of proteins belonging to the “apoptosis- and splicing-associated protein complex”, potentially influencing the alternative splicing process and consequently leading to a possible susceptibility to programmed cell death. These findings indicate that modifying natural AMPs may be a promising strategy for developing selective anticancer drugs and pinpoint Myr-B as an interesting target for future studies.

## 1. Introduction

Antimicrobial peptides (AMPs) are natural bioactive peptides produced by all organisms as key innate immune components able to defend them against a broad spectrum of pathogens (bacteria, viruses, fungi and parasites) [1]. AMPs include peptides from various sources in the natural environment: in particular, marine and terrestrial animals are considered an important reservoir of active AMPs [2]. Interestingly, the innate immune system of organisms such as fish or insects, living in different but hostile high-microbial-load environments, significantly relies on AMPs [2]. Unlike insects, which lack an adaptive innate immune system and use AMPs as the primary and most important effectors of their immune system, most fish AMPs also act as modulators of adaptive immunity [3,4].

Moreover, AMPs can be classified based on their amino acid composition, charge, structures and biological activities [5]. Their variability in origin (from plants to insects, microbes and animals), mode of action (from membrane disruption to intracellular targeting) and activity (antibacterial, antiviral, antifungal, antiparasitic, anticancer) make AMPs useful in a variety of fields, from biotechnology to pharmaceutics. AMPs are primarily known for their antibacterial action: they exhibit a broad spectrum of activity against several non-human pathogens, thus generating considerable interest in their potential applications in agriculture, animal health and food preservation. Specifically, AMPs could play a key role in counteracting pathogens that affect plant and livestock health and food safety, thus improving agricultural productivity and sustainability [6]. On the other hand, AMPs represent a promising therapeutic option against multidrug-resistant infections due to their unique ability to combat human pathogenic microorganisms [7,8]. Their mode of action involves destabilizing, disintegrating or forming pores in the microbial membrane, with little chance of the pathogens developing resistance [9]. Non-membranolytic mechanisms acting via cellular targets are less studied but still relevant: some AMPs can cross the membrane, penetrate the cell and interact with DNA/RNA to inhibit protein synthesis and with intracellular proteins/enzymes to block cellular replication [10,11]. AMPs could therefore replace or be used in synergistic combinations with conventional antibiotics, counteracting antimicrobial resistance, which represents a global health crisis [12,13].

AMPs have also been investigated as anticancer agents capable of overcoming the main limitations of current cancer therapies. Cancer remains one of the most life-threatening diseases worldwide and is characterized by the uncontrolled growth and proliferation of abnormal cells following the accumulation of genomic and molecular alterations, including inherited mutations, epigenetic changes and DNA damage caused by harmful agents [14]. Although conventional cancer therapies (chemotherapy, radiotherapy and surgery), which have been significantly improved and integrated with new approaches, remain the mainstay treatment for many types of cancer, some biomedical needs in cancer management remain unmet. Conventional chemotherapy, however, affects normal cells, causing toxic side effects that severely compromise patients’ immune systems and organs [15]. In addition, following an initial positive response to chemotherapy, a resistance to treatment may unfortunately develop due to the high adaptability of cancer cells through the following main mechanisms: (i) genomic instability leading to the formation of clones with varying sensitivities to drugs; (ii) epigenetic reprogramming altering gene expression and inducing phenotypic plasticity; (iii) alteration of drug efflux and metabolism modifying pharmacokinetics and bioavailability; (iv) escape from cell death (apoptosis resistance) and/or immune evasion; and (v) metabolic reprogramming promoting cancer cell survival under stress conditions and during chemotherapy [16,17,18,19]. These key mechanisms are responsible for cancer progression, recurrence and poor patient prognosis [20]. These challenges highlight the urgent need to investigate new therapeutic molecules that are selective towards target cancer cells and are thus likely capable of reducing the side effects of chemotherapy and/or overcoming the phenomenon of tumor resistance [21,22]. Cationic AMPs have received widespread attention as anticancer peptides (ACPs) because of their ability to selectively interact with the negatively charged surfaces of cancer cell membranes, with mechanisms of action that significantly reduce the likelihood of cancer cells developing resistance [5,23]. Similarly to AMPs’ action on bacterial membranes, ACPs disrupt cancer cell membranes by forming pores through the barrel-stave model, toroidal model or carpet model, thus leading to cell lysis and death [2]. The membrane-lytic effect of ACPs is likely enhanced by the specific composition and characteristics of cancer cell membranes compared to those of normal cells [24]. Unlike healthy cell membranes, cancer cells exhibit the following specific membrane characteristics that may increase their vulnerability to ACPs: (i) increased negative charge (presence of phosphatidylserine, O-glycosylated mucins and heparin sulfate on the outer leaflets of the plasma membrane); (ii) increased membrane fluidity (reduced cholesterol levels); and (iii) increased surface area (abundant filopodia and microvilli) [25,26]. In addition to membrane-disruptive mechanisms, ACP action in cancer cells can be mediated by intracellular targets, thus inducing tumor apoptosis and necrosis, regulating tumor angiogenesis and immunity and inhibiting tumor cell growth and motility [2]. Several ACPs from different sources have been proven to interact with cancer cell membranes and/or regulate intracellular signaling pathways, such as LL-37, defensins and lactoferricin from mammals; magainin 2 from amphibians; melittin, cecropins and AMP fractions from insects [4,26,27,28,29]. Marine AMPs, such as fish-derived pardaxin and epinecidin-1, have also been shown to possess anticancer properties [30,31]. Moreover, ACPs can be synthetically produced, as with SVS-1, KLAK and D-K6L9 peptides, and therefore rationally modified to enhance their cancer target specificity [32,33]. In this study, we selected Antarctic fishes as an ACP source. Living in a very extreme environment, these species have evolved specific adaptations and produce bioactive molecules, including AMPs, that can differ significantly from those isolated from fish inhabiting temperate waters [34,35,36].

In particular, we focused on a natural AMP called chionodracine (Cnd) from the Antarctic icefish *Chionodraco hamatus* [34,37]. Based on its scaffold, we successfully designed peptide mutants effective against human pathogens [38]. In particular, the natural peptide Cnd sequence (22 amino acids) was modified by increasing the number of charged residues. One of the mutants, named KHS-Cnd, in which histidines and serines were all replaced by lysines, was highly active against ESKAPE bacteria [39], whereas shorter forms derived from it with an N-terminal lipid tail of myristic acid were active against fungal pathogens [40]. Myristoylation is one of the most commonly used modifications for enhancing the biological activity of natural AMPs [41]. To the best of our knowledge, families of frequently myristoylated (myr) AMPs with enhanced anticancer activity include cecropin derivatives, such as the CM4 peptide, or defensin fragments, while PMAP peptides are the most well-known antibacterial myr AMP [42,43,44]. Unlike these existing myr AMPs, KHS-Cnd-derived myr peptides, considered here, were developed using a rational modular design approach starting from the fragmentation of parent AMPs, thus obtaining three very short (11 amino acids) but potent antifungal lipopeptides, labeled Myr-A, Myr-B and Myr-C. Notably, the corresponding non-myr peptides (Pep-A, Pep-B and Pep-C) showed little antifungal activity [40].

In this study, we investigated the anticancer potential of these short KHS-Cnd-derived myr peptides compared to the action of the corresponding non-lipidated peptides. The rationale for focusing on the anticancer activity of these myr peptides is based on previous findings showing their ability to interact with synthetic biological membranes containing negative charges by adopting an extended conformation parallel to the lipid surface. They are therefore potentially selective towards cancer membranes, as they have also shown low cytotoxicity and low hemolysis against human fibroblasts and rabbit erythrocytes, respectively [40].

First, we determined their cytotoxicity against the HeLa cancer cell line and tried to evaluate the modifications caused in cell membranes and the type of induced cell death. Next, to assess their selectivity, the cytotoxic effect was also evaluated against two types of primary human fibroblasts. Moreover, an additional cancer cell line, namely Caco-2 cells, was tested to evaluate the cancer cell specificity of the Myr-B peptide, selected as the best-performing/most promising ACP against HeLa cells. Finally, we performed a label-free quantification (LFQ) proteomics analysis to deepen our understanding of the molecular changes produced as a result of the interaction of the peptide with the HeLa cancer cell membrane. Our results highlight that modifying natural AMPs may be a promising strategy for developing selective ACPs and provide additional evidence on the physiological response of cancer cells “attacked” by AMPs.

## 2. Results

### 2.1. Cytotoxic Effect of Lipopeptides on Human Cancer Cells

**Figure 1:**
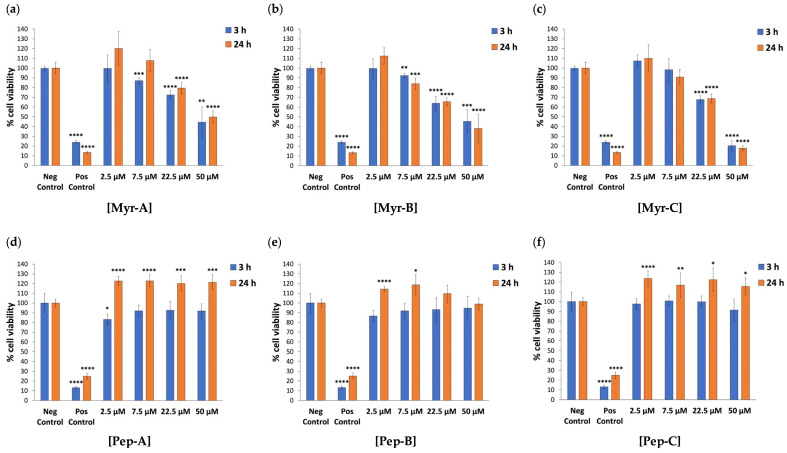
The cytotoxic effect of N-myristoylated peptides against human cervical cancer HeLa cells. Cell viability was assessed using MTT assay for HeLa cell line after 3 h and 24 h of incubation with N-myristoylated Myr-A (**a**), Myr-B (**b**) and Myr-C (**c**) peptides and corresponding non-myristoylated Pep-A (**d**), Pep-B (**e**) and Pep-C (**f**) ones. Data are expressed as a percentage of viable cells in the presence of different peptide concentrations (2.5–50 µM) compared to untreated HeLa cells (negative control). Positive control represents cells treated with 2% NaN_3_. Untreated cells serve as negative control. All experiments were performed using two independent replicates, each with at least three repeats. Statistical significance was assessed using one-way ANOVA followed by Dunnett’s post hoc test (GraphPad Prism 11.0). Difference from the negative control was considered statistically significant as follows: * adjusted *p*-value < 0.05; ** *p*-value < 0.01; *** *p*-value < 0.001; **** *p*-value < 0.0001.

The cytotoxic effect of N-myristoylated (Myr-A, Myr-B and Myr-C) and corresponding non-myristoylated (Pep-A, Pep-B and Pep-C) peptides was evaluated against the human cervical cancer HeLa cell line using the MTT assay after 3 h and 24 h of exposure to increasing peptide concentrations (0, 2.5, 7.5, 22.5 and 50 µM). Based on their predicted ability to interact with negatively charged cell membranes [40], their cytotoxicity was assessed over a short period of time (3 h) and after one cell cycle (24 h) to evaluate rapid and acute effects, respectively, thus avoiding any potential loss of peptide stability over time due to the degradative action of proteases in the cell culture medium. Overall, the bar graphs show a dose-dependent decrease in HeLa cell viability after 3 h and 24 h exposure to myr peptides (Figure 1a–c). Sodium azide (NaN_3_), widely used to induce mitochondrial dysfunction and cytotoxicity in cell viability assays [45,46], was employed as a positive control for cell death at a high concentration (2% *w*/*v*) to ensure the maximum and reproducible loss of cell viability through the rapid arrest of cellular metabolic activity [47]. Moreover, pairwise comparisons against untreated HeLa cells (negative control) showed statistically significant differences after 3 h and 24 h exposure only at concentrations of 22.5 µM and 50 µM for all three myr peptides (Figure 1a–c and Supplementary Table S1). In particular, the cell viability of HeLa cells at 3 h and 24 h exposure to myr peptides at a concentration of 22.5 µM ranged between 64% and 79% compared to the negative control, whereas, at a peptide concentration of 50 µM after 3 h of exposure, cell viability reached values of 44%, 45% and 20% for Myr-A, Myr-B and Myr-C, respectively. A similar statistically significant trend was confirmed after 24 h of exposure to all three myr peptides at a 50 µM concentration, thus highlighting the strong cytotoxic effect on HeLa cells at the highest tested dose.

As for non-myr peptides, pairwise comparisons against the negative control showed no statistically significant differences in the cell viability of HeLa cells after 3 h of exposure to all peptide concentrations (Figure 1d,e and Supplementary Table S1). Overall, the 3 h exposure of HeLa cells to non-myr peptides did not result in any cytotoxic effect, even at the highest doses of all peptides, whereas a statistically significant proliferative effect was observed after 24 h of exposure to all concentrations of non-myr peptides, except for 22.5 µM and 50 µM of Pep-B (Figure 1d,e).

**Figure 2:**
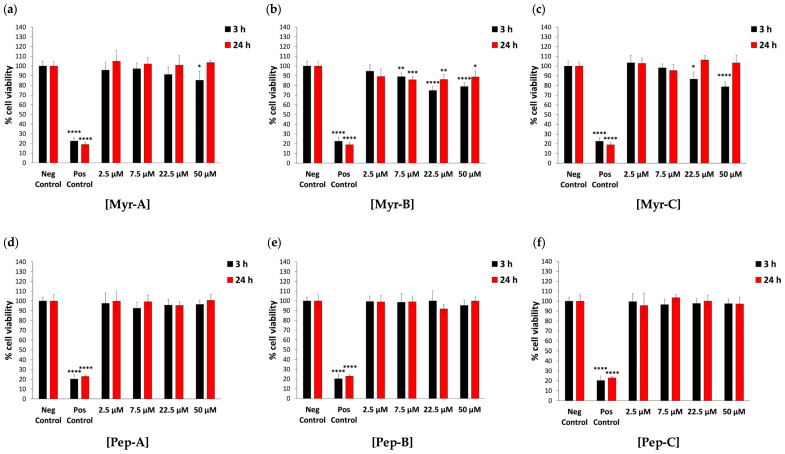
The cytotoxic effect of N-myristoylated peptides against primary human FB789 fibroblasts. Cell viability was assessed using the MTT assay for the FB789 cell line after 3 h and 24 h of incubation with N-myristoylated Myr-A (**a**), Myr-B (**b**) and Myr-C (**c**) peptides and corresponding non-myristoylated Pep-A (**d**), Pep-B (**e**) and Pep-C (**f**) ones. Data are expressed as the percentage of viable cells in the presence of different peptide concentrations (2.5–50 µM) compared to untreated FB789 cells (negative control). Positive control represents cells treated with 2% NaN_3_. Untreated cells serve as negative control. All experiments were performed using two independent replicates, each with at least three repeats. Statistical significance was assessed using one-way ANOVA followed by Dunnett’s post hoc test (GraphPad Prism 11.0). Difference from the negative control was considered statistically significant as follows: * adjusted *p*-value < 0.05; ** *p*-value < 0.01; *** *p*-value < 0.001; **** *p*-value < 0.0001.

**Figure 3:**
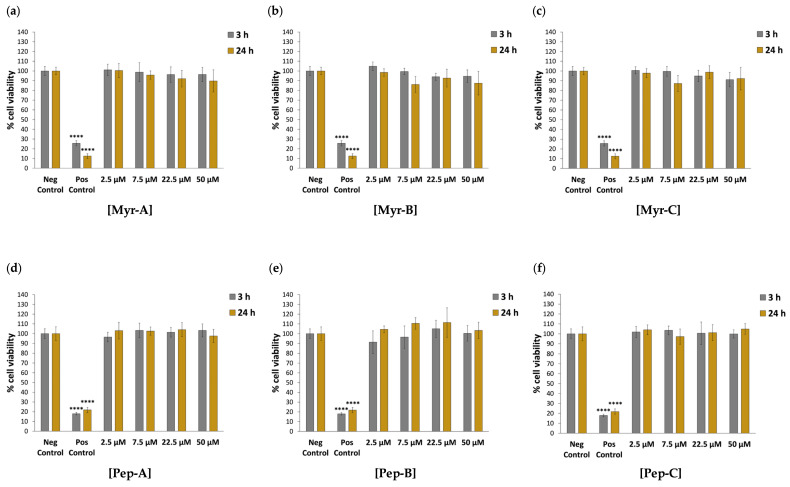
The cytotoxic effect of N-myristoylated peptides against primary human fibroblasts (HDF). Cell viability was assessed using the MTT assay for the HDF cell line after 3 h and 24 h of incubation with N-myristoylated Myr-A (**a**), Myr-B (**b**) and Myr-C (**c**) peptides and corresponding non-myristoylated Pep-A (**d**), Pep-B (**e**) and Pep-C (**f**) ones. Data are expressed as a percentage of viable cells in the presence of different peptide concentrations (2.5–50 µM) compared to untreated FB789 cells (negative control). Positive control represents cells treated with 2% NaN_3_. Untreated cells serve as negative control. All experiments were performed using two independent replicates, each with at least three repeats. Statistical significance was assessed using one-way ANOVA followed by Dunnett’s post hoc test (GraphPad Prism 11.0). Difference from the negative control was considered statistically significant as follows: **** adjusted *p*-value < 0.0001.

To assess whether the observed cytotoxic activity of myr peptides may be selective for cancer cells, normal human cell lines represented by human primary fibroblasts FB789 and human primary dermal fibroblasts (HDFs) were treated with the same procedure used for HeLa cells. The bar graphs in Figure 2 and Figure 3 show the cell viability of FB789 and HDF, respectively, after 3 h and 24 h exposure to the three N-myr and corresponding non-myr peptides at the same concentrations tested against HeLa cells. In particular, at 22.5 and 50 µM, Myr-A, Myr-B and Myr-C peptides did not exhibit the cytotoxicity observed for cancer cells. The lowest percentage of FB789 cell viability was observed after 3 h of exposure to 22.5 and 50 µM Myr-B (75% and 79%, respectively) compared to the negative control (Figure 2a–c and Supplementary Table S2). At this treatment time, in fact, a slight dose-dependent effect for all three myr peptides was evident, although not always statistically significant (Figure 2a–c and Supplementary Table S2). After 24 h of exposure, however, no dose-dependent cytotoxic effect was observed, thus confirming the data previously reported by Bugli and colleagues, who did not consider the early exposure time (3 h) [40].

Moreover, at 3 h and 24 h, FB789 cells treated with non-myr peptides did not show any statistically significant cytotoxic effect. Even at the highest tested dose, for all peptides, the cell viability was >90% (Figure 2d,e).

In addition to FB789 cells, we also tested the cytotoxicity of myr and non-myr peptides against HDF cells, representing other normal primary fibroblasts from a different tissue than FB789. Regarding HDF cell viability after 3 h and 24 h of peptide exposure, no percentage values lower than 87% were found for any of the six studied peptides (Figure 3 and Supplementary Table S3), thus confirming the selective action of myr peptides against HeLa cells.

As reported in Table 1, the calculated IC_50_ values for the HeLa cells of all myr peptides at both exposure time points were close to the highest tested dose or between the concentrations of 22.5 µM and 50 µM. Specifically, Myr-A exhibited the highest IC_50_ values, followed by Myr-B and then Myr-C (Table 1).

However, in our previous study [40], 50 μM Myr-C peptide showed a 28.5% hemolytic effect on rabbit erythrocytes, while, at the same concentration, the Myr-A and Myr-B peptides had a hemolytic effect of less than 7% [40]. Therefore, the hemolysis data, together with the data on cell viability, led to the selection of the Myr-B lipopeptide for subsequent analyses, as it is less hemolytic than Myr-C and more cytotoxic than Myr-A against HeLa cells.

**Figure 4:**
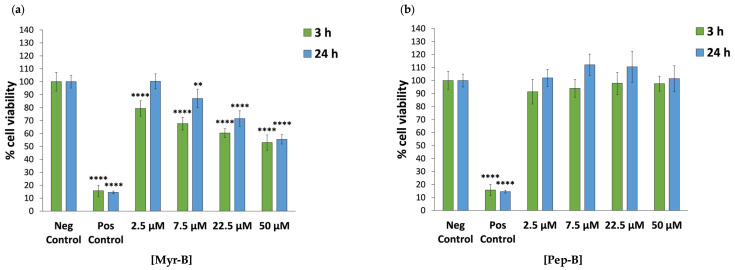
The cytotoxic effect of Myr-B peptide against human colon cancer Caco-2 cells. Cell viability was assessed using the MTT assay for the Caco-2 cell line after 3 h and 24 h of incubation with N-myristoylated Myr-B peptide (**a**) and corresponding non-myristoylated Pep-B peptide (**b**). Data are expressed as a percentage of viable cells in the presence of different peptide concentrations (2.5–50 µM) compared to untreated Caco-2 cells (negative control). Positive control represents cells treated with 2% NaN_3_. Untreated cells serve as negative control. All experiments were performed using two independent replicates, each with at least three repeats. Statistical significance was assessed using one-way ANOVA followed by Dunnett’s post hoc test (GraphPad Prism 11.0). Difference from the negative control was considered statistically significant as follows: ** adjusted *p*-value < 0.01; **** *p*-value < 0.0001.

Firstly, the cytotoxicity of the Myr-B peptide, selected as the most effective/promising ACP in HeLa cells, was evaluated on an additional cancer cell line, namely human colon cancer Caco-2 cells. As reported in Figure 4a, the MTT assay showed a dose-dependent decrease in Caco-2 cell viability after 3 h and 24 h of exposure to the Myr-B peptide. As for the corresponding non-myr peptide, pairwise comparisons against the negative control showed no statistically significant differences in the viability of Caco-2 cells after either 3 h or 24 h of exposure to Pep-B at all concentrations considered (Figure 4b and Supplementary Table S4). Conversely, pairwise comparisons against untreated Caco-2 cells (negative control) showed statistically significant differences in cell viability after 3 h of exposure to all Myr-B concentrations and the same after 24 h, except for the lowest Myr-B dose tested (2.5 µM, Supplementary Table S4). Specifically, Caco-2 cell viability after 3 h of exposure to the Myr-B peptide at increasing concentrations (2.5–50 µM) ranged from 79% to 53% compared to the negative control, whereas, after 24 h of exposure, cell viability ranged from 100% to 55%. As expected, the Myr-B peptide IC_50_ values (48.88 ± 0.86 µM and 51.07 ± 0.13 µM) calculated for Caco-2 cells after 3 h and 24 h of exposure, respectively, were higher than those determined for HeLa cells. By testing an additional cancer cell line, we observed that HeLa cells are more sensitive to the Myr-B peptide than Caco-2 cells, thus highlighting a cancer cell type-specific selectivity for the chosen ACP.

### 2.2. Assessment of Myr-B Peptide-Induced Cell Membrane Damage and Morphological Changes on Human Cancer Cells

**Figure 5:**
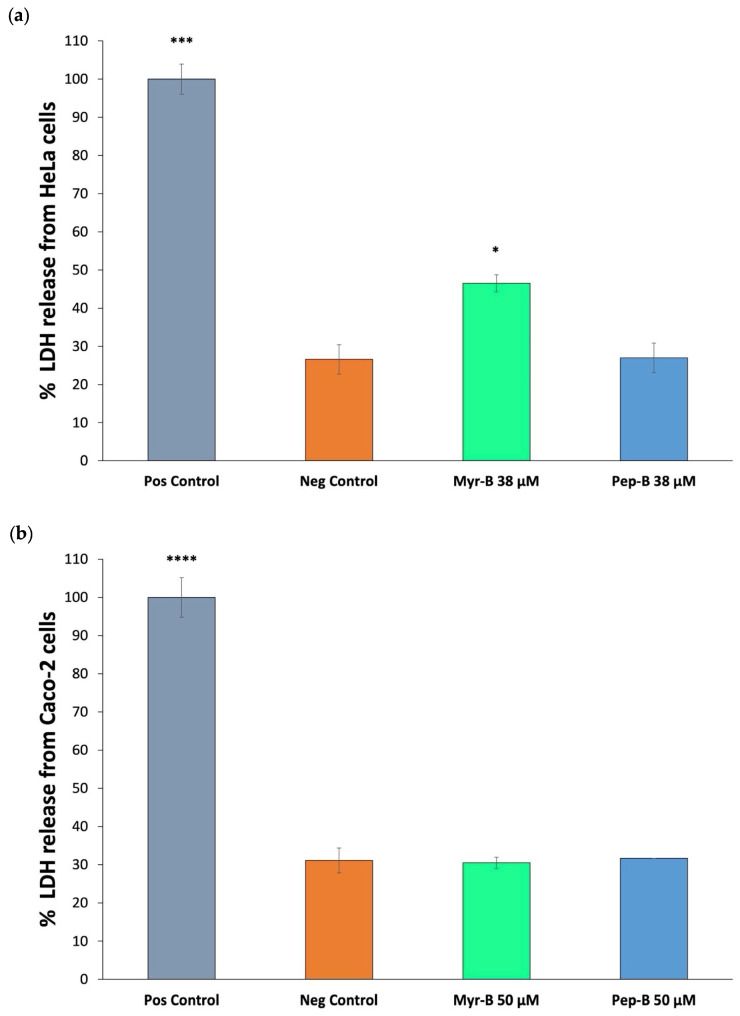
Evaluation of Myr-B peptide-induced death of human cervical cancer HeLa (**a**) and colon cancer Caco-2 (**b**) cells. Cell membrane integrity was assessed through lactate dehydrogenase (LDH) release from untreated HeLa or Caco-2 cells (negative control) after 3 h exposure of HeLa and Caco-2 cells to their respective IC_50_ doses of Myr-B peptide (38 μM and 50 μM, respectively) and from HeLa or Caco-2 cells treated with a lysis solution to release all LDH (positive control). The exposure to Pep-B was carried out under the same conditions as the corresponding treatment with the Myr-B peptide for each cancer cell line. Statistical significance was assessed using one-way ANOVA followed by Dunnett’s post hoc test (GraphPad Prism 11.0). Difference from the negative control was considered statistically significant as follows: * adjusted *p*-value < 0.05; *** *p*-value < 0.001; **** *p*-value < 0.0001.

To evaluate the consequences of the cytotoxicity induced by the Myr-B peptide, we selected different methodologies able to provide information on cell membrane damage (loss of barrier function) and morphological changes (structural alterations). First, the activity of the enzyme lactate dehydrogenase (LDH) released into the cell culture medium was measured as an indicator of impaired cell membrane integrity. Although the LDH assay is not specific for either apoptosis or necrosis—the major processes of cell death—in combination with other methods, it can be useful for detecting necrotic cells, whose key feature is the damage of the cell membrane [48]. After the 3 h exposure of HeLa cells to the IC_50_ dose of the Myr-B peptide (38 μM), LDH levels increased significantly, with a value almost twice that measured in the untreated cells (negative control, Figure 5a and Supplementary Table S5). The levels of LDH in the culture medium did not differ significantly between 38 μM Pep-B-treated and untreated cells, confirming that the HeLa cell membrane was damaged after exposure to the Myr-B peptide, but not after treatment with the corresponding unmodified peptide (Figure 5a).

In contrast, after the 3 h exposure of Caco-2 cells to the IC_50_ dose of the Myr-B peptide (50 μM), LDH levels in the culture medium did not differ significantly from those measured in either untreated cells (negative control) or in 50 μM Pep-B-treated Caco-2 cells (Figure 5b and Supplementary Table S5). This latter result showed that the Myr-B peptide does not impair the integrity of the Caco-2 cell membrane, unlike HeLa cells, thus confirming the susceptibility of HeLa cells to Myr-B, likely determined through the different modes of peptide–membrane interaction.

**Figure 6:**
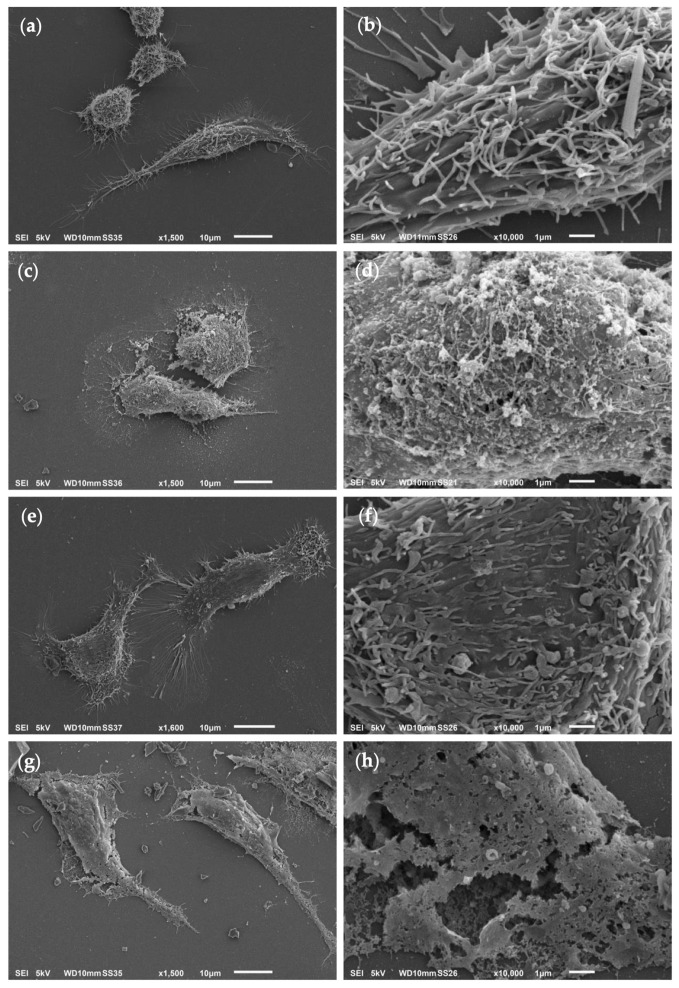
Scanning electron microscopy (SEM) analysis of Myr-B peptide-induced effects on human cervical cancer HeLa cells. SEM micrographs (acquired and processed using JEOL inTouchScope Interface software, https://www.jeol.com/products/scientific/sem/JSM-IT510.php, access date: 23 April 2026) of untreated HeLa cells (negative control) (**a**,**b**), HeLa cells after 3 h exposure to 38 μM Myr-B (**c**,**d**) and 38 μM Pep-B (**e**,**f**) and HeLa cells treated with 2% NaN_3_ (positive control) (**g**,**h**). Magnification and scale bars = (**a**–**d**) 1500× and 10 μm, respectively; (**e**–**h**) 10,000× and 1 μm, respectively.

To assess Myr-B peptide-induced effects on cell morphology, scanning electron microscopy (SEM) analysis was performed on human cervical cancer HeLa cells. These cells were exposed for 3 h to 38 μM Myr-B peptide and compared to untreated cells (negative control), Pep-B-treated cells and those treated with 2% NaN_3_ (positive control, Figure 6).

Untreated HeLa cells appeared as spindle-shaped cells with a series of cytoplasmic protrusions (Figure 6a,b). After 3 h of exposure to 38 μM Myr-B peptide, HeLa cells exhibited morphological changes, including a slightly rounded shape due to cell swelling and a reduction/absence of protrusions (Figure 6c,d). Moreover, HeLa cells treated with Myr-B showed severely damaged membranes with leakage of intracellular material. In contrast, HeLa cells treated for 3 h with 38 μM Pep-B peptide maintained a similar morphology to that of untreated cells (Figure 6e,f), with slight differences in cell surfaces compared to the negative control. However, these Pep-B-induced alterations were not comparable to those induced by the corresponding myr peptide and did not lead to cell death, as confirmed by the cytotoxicity data. Hence, the SEM analysis of HeLa cells treated with the Myr-B peptide revealed an advanced state of necrosis compared to the negative control, although not as extensive as in the positive control (Figure 6g,h).

**Figure 7:**
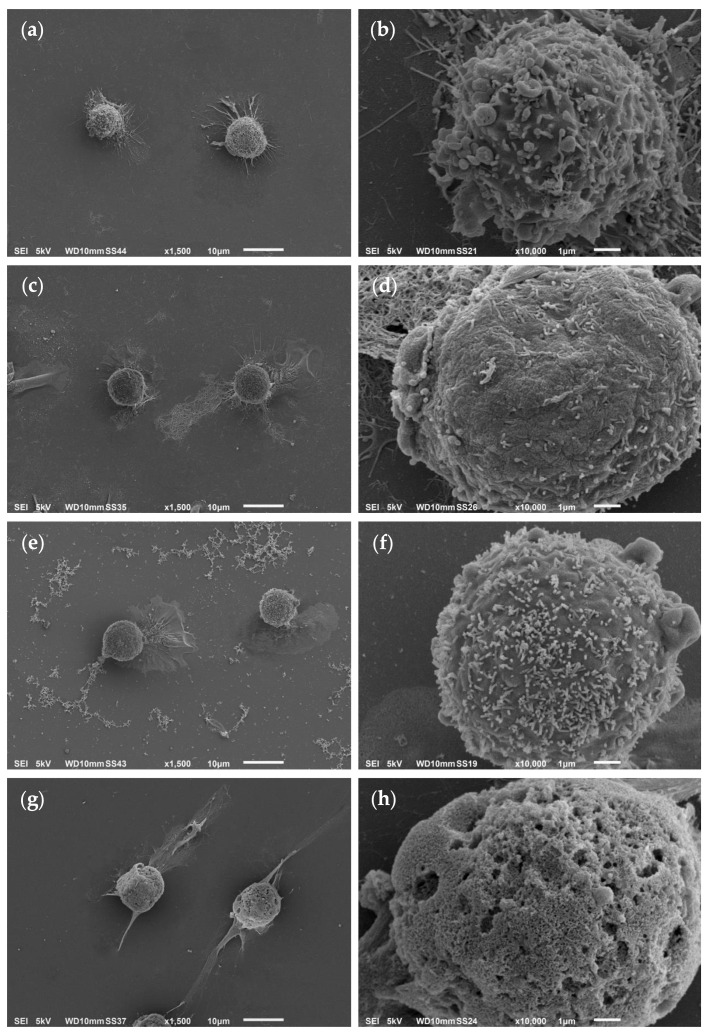
SEM analysis of Myr-B peptide-induced effects on human colon cancer Caco-2 cells. SEM micrographs (acquired and processed using JEOL inTouchScope Interface software) of untreated Caco-2 cells (negative control) (**a**,**b**), Caco-2 cells after 3 h exposure to 50 μM Myr-B (**c**,**d**) and 50 μM Pep-B (**e**,**f**) and Caco-2 cells treated with 2% NaN_3_ (positive control) (**g**,**h**). Magnification and scale bars = (**a**–**d**) 1500× and 10 μm, respectively; (**e**–**h**) 10,000× and 1 μm, respectively.

To further evaluate the specific selectivity of the Myr-B peptide towards HeLa cells, SEM analysis was also performed on human colon cancer Caco-2 cells to thoroughly explore Caco-2 cell membrane integrity, as suggested by the relative LDH assay. To achieve this, Caco-2 cells were exposed for 3 h to the IC_50_ dose of the Myr-B peptide and compared with untreated cells (negative control), 50 μM Pep-B-treated cells and those treated with 2% NaN_3_ (positive control, Figure 7).

Untreated Caco-2 cells showed the characteristic morphology of immature epithelial cells not yet fully polarized but perfectly adherent: lamellipodia and thin filopodia were evident at the cell margins, indicating proliferative activity and a dynamic reorganization of the cortical cytoskeleton (Figure 7a). Furthermore, as shown in the magnified image in Figure 7b, the surface of the negative control appeared finely granular, with short cytoplasmic protrusions irregularly distributed and likely attributable to immature microvilli or membrane microextensions.

After 3 h of exposure to 50 μM of the Myr-B and Pep-B peptides, both treated Caco-2 cells showed morphological changes on their surface compared with untreated cells (Figure 7c–f), mainly associated with a reduction in protrusions, sporadic blebbing and a slight loss of adhesion, which were in no way comparable to those observed in the highly necrotic positive control (Figure 7g,h). The reduction in superficial protrusions was certainly more evident in the Caco-2 cells treated with Myr-B rather than the Pep-B peptide. Interestingly, unlike Myr-B-treated HeLa cells, Caco-2 cells showed no cell membrane damage nor any leakage of intracellular material after exposure to Myr-B, in perfect agreement with the LDH data.

### 2.3. Label-Free Proteomics of Human Cancer HeLa Cells Treated with Myr-B Peptide

**Figure 8:**
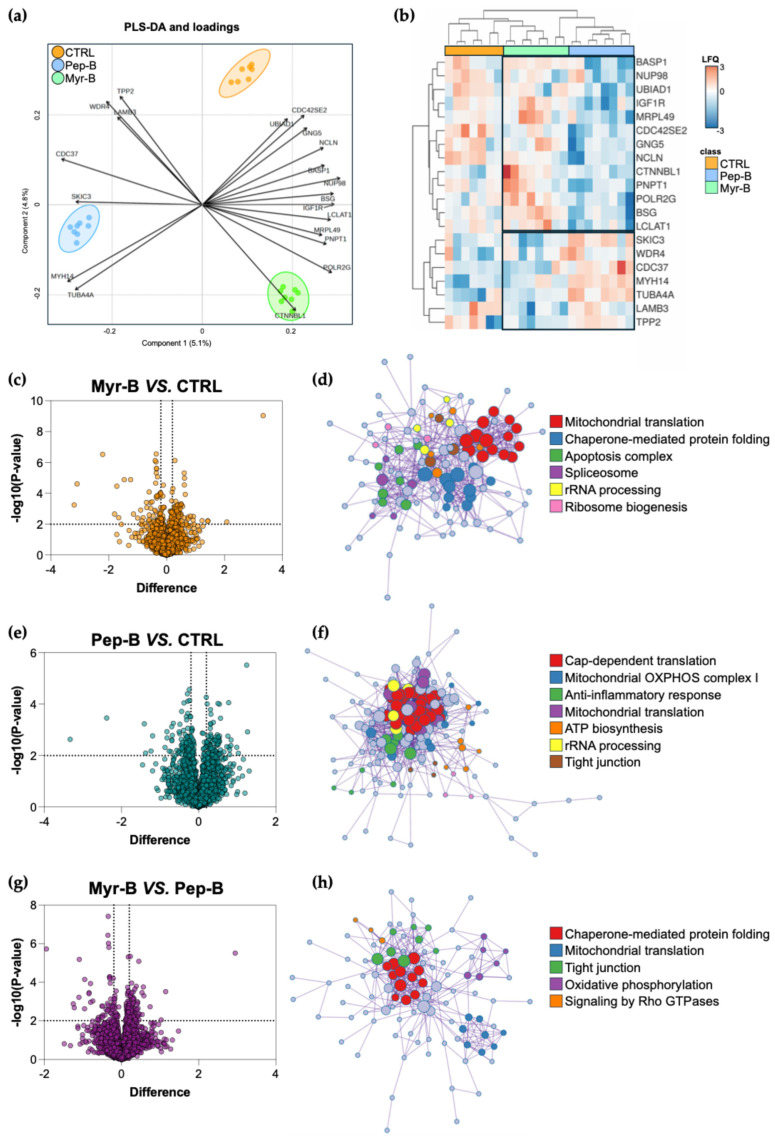
Proteomic analysis of human cervical cancer HeLa cells treated with Myr-B and Pep-B peptides. Biplot of PLS-DA showing group separation and the top 20 feature loadings contributing to the variance along the principal components were obtained using MetaboAnalyst 6.0 (**a**). The selected proteins were clustered in a heatmap according to their LFQ values in the three experimental conditions; boxes were used to highlight different abundance patterns in Myr-B and Pep-B samples (**b**). Volcano plot analysis was performed with GraphPad Prism 11.0 using multiple unpaired *t*-tests with Welch correction to identify differentially regulated proteins, with a relative PPI network and functional enrichment determined using Metascape for the Myr-B vs. CTRL (**c**,**d**), Pep-B vs. CTRL (**e**,**f**) and Myr-B vs. Pep-B (**g**,**h**) comparisons, respectively. Differential proteins were selected with a difference cutoff ± 0.2 and −log10 *p*-value > 2.

To evaluate the broader impact of Myr-B and Pep-B on the HeLa cell proteome, we performed a label-free quantification (LFQ) proteomics analysis. Following appropriate filtering and data processing, we identified approximately 4200 proteins in the global LC-MS/MS dataset that included the sample replicates of the three studied groups: 38 μM Myr-B- or Pep-B-treated cells for 3 h and control (CTRL) cells. Partial Least Squares Discriminant Analysis (PLS-DA) demonstrated a clear separation of the three groups along the selected principal components, showing a variance of 5.1% and 4.8% for components 1 and 2, respectively (Figure 8a). The PLS-DA model was validated through cross-validation and demonstrated a discrete predictive ability (Q2 = 0.39), good fitness (R2 = 1.0) and an accuracy of 0.8. Moreover, the top 20 features contributing to group variance were identified from the PLS-DA loadings (Figure 8a).

The LFQ abundance profiles of these 20 proteins were further analyzed through hierarchical clustering. As shown in Figure 8b, the selected proteins displayed distinct abundance patterns in Myr-B- and Pep-B-treated cells, suggesting that they significantly contribute to the observed proteomic differences. Notably, several of these proteins, such as BSG, CDC37, CTNNBL1, IGF1R and NUP98, are functionally linked to processes governing signal transduction, RNA metabolism, nucleocytoplasmic transport and translational control, including species with recognized roles in cancer-associated regulatory networks. This functional convergence supports the biological relevance of the clustering output, suggesting a coherent molecular variation associated with peptide treatment.

Quantitative volcano plot analysis revealed a subset of 139 significantly regulated proteins in the Myr-B vs. CTRL comparison, with 79 of them being upregulated and 60 being downregulated (Figure 8c and Supplementary Table S6). These proteins formed a protein–protein interaction (PPI) network, enriching processes related to mitochondrial translation, protein folding, RNA processing and apoptotic pathways (Figure 8d). In contrast, Pep-B-treated cells showed 184 differentially abundant proteins compared with the CTRL, including 93 up- and 91 downregulated species (Figure 8e and Supplementary Table S7). PPI and functional enrichment analysis highlighted the effects on RNA processing, anti-inflammatory response and mitochondrial metabolism, including translation, oxidative phosphorylation (OXPHOS) and ATP production (Figure 8f). Finally, a direct comparison of Myr-B and Pep-B treatments identified 128 differential proteins, of which 80 were more abundant and 48 were less abundant in Myr-B relative to Pep-B (Figure 8g and Supplementary Table S8). Functional enrichment revealed pathways shared by both peptides, particularly those associated with mitochondrial translation and OXPHOS (Figure 8h). This approach enabled a preliminary characterization of differentially expressed proteins across the three experimental groups. Importantly, as these candidate proteins were not derived from multiple-testing-corrected analyses, they should be interpreted as exploratory and hypothesis-generating.

**Figure 9:**
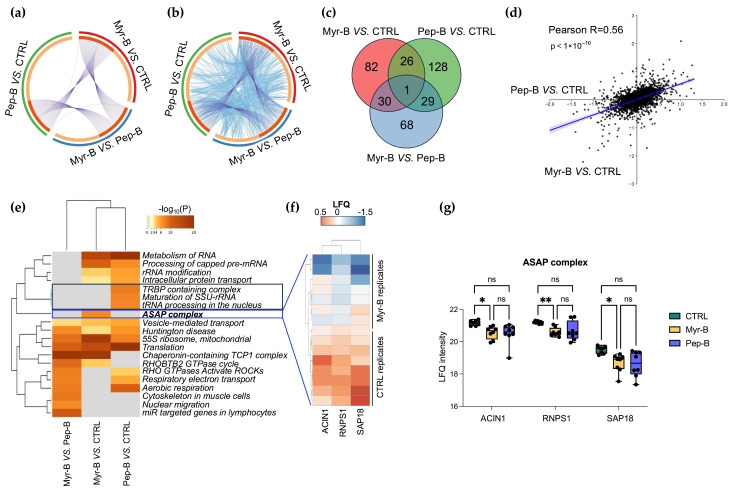
Bioinformatic and multi-comparison analysis of proteomics data. Circos plots were used to identify overlaps between genes (**a**) and shared ontology terms (**b**) in the three pairwise comparisons. Venn analysis was performed to capture qualitative differences or similarities in the hits identified through proteomics data analysis (**c**). Pearson correlation analysis was carried out using the difference values for Myr-B vs. CTRL and Pep-B vs. CTRL comparisons to calculate the correlation R value (**d**). Heatmap of enriched ontology terms across input gene lists from Myr-B, Pep-B and CTRL conditions, colored according to *p*-values (top 20 clusters); blank cells indicate a lack of enrichment for that term in the corresponding gene list (**e**). Heatmap of LFQ intensity values of the core members of the ASAP complex in CTRL and Myr-B cell replicates was obtained by applying unit variance scaling to rows and correlation distance and average linkage to both rows and columns (**f**). Statistical validation of ASAP complex member under-expression in Myr-B-treated samples. Two-way ANOVA was used for multiple comparisons based on the Tukey test (GraphPad Prism 11.0). * Adjusted *p*-value < 0.05; ** *p*-value < 0.01; ns: not significant (**g**).

To further dissect similarities and differences in the proteomic responses to the two peptides, we carried out a multi-comparison analysis. While overlaps were observed among the differential protein lists from the three pairwise comparisons, both at the gene and at the enriched ontology levels (Figure 9a,b), these qualitative intersections were not indicative of common regulatory trajectories (Figure 9c and Supplementary Table S9), whereas quantitative correlation analyses uncovered distinct proteomic profiles. Indeed, as shown in Figure 9d, Pearson correlation between the differential proteomes of Myr-B vs. CTRL and Pep-B vs. CTRL revealed only a limited correlation (Pearson R = 0.56, *p* < 1 × 10^−10^), supporting the idea that the two peptides modulate cellular pathways through largely distinct mechanisms. Notably, heatmap visualization of enriched biological processes across the three comparisons identified a subset of terms shared by both treatments, mainly related to RNA metabolism, translation and transport. In contrast, Pep-B selectively impacted pathways involved in miRNA biogenesis, rRNA maturation and tRNA processing, whereas Myr-B uniquely enriched functions linked to cell death control (Figure 9e). Of particular relevance, enrichment of the ASAP (apoptosis- and splicing-associated protein) complex was accompanied by the coherent down-modulation of its three core subunits (RNPS1, ACIN1 and SAP18) in Myr-B-treated cells relative to the controls (Figure 9f). To statistically validate the selective under-expression of ASAP components in Myr-B-treated, but not in Pep-B-treated, HeLa cells, we performed an ANOVA-based multiple comparison against control cells. As already demonstrated, all members (ACIN1, RNPS1 and SAP18) consistently showed reduced levels in Myr-B-treated samples based on LFQ intensities (Figure 9g).

## 3. Discussion

While conventional chemotherapy targeting/killing fast-growing cancer cells continues to be the main strategy for cancer management, the development of next-generation anticancer drugs capable of overcoming the limitations of traditional cancer therapies related to toxicity and drug resistance represents a priority at present. Current treatments, in fact, are non-specific, as their mechanisms of action do not discriminate against other fast-growing cells, thus affecting not only cancer cells but also healthy ones [49]. In addition, the development of tumor resistance, classified as intrinsic (or primary) or acquired chemoresistance based on the occurrence timeline, is a major obstacle in cancer therapy. These forms of resistance are driven by various and complex molecular mechanisms, among which genetic/epigenetic factors or escape from apoptotic programmed cell death represent only some of the underlying causes [50,51,52].

In light of these issues, discovering new therapeutic options that specifically target cancer cells while sparing normal cells is a key global health mission. In this context, exploring different anticancer options, including small-molecule targeted drugs such as peptides, is emerging as a significant research field [53]. Anticancer peptides (ACPs) are peptide-based anticancer agents generally composed of 5–50 amino acids that are mostly cationic and amphipathic [5]. Several ACPs have been developed based on natural antimicrobial peptides (AMPs), since some AMPs also exhibit anticancer properties [54]. The first advantage of designing ACPs is that their production uses well-established solid-phase peptide synthesis methods [55]. Chemical modifications (amino acid incorporation/substitution, cyclization, PEGylation and lipidation) are well-documented for these molecules and are available for enhancing their pharmacokinetics and stability [56]. Moreover, when designing new ACPs, it is necessary to consider that the mechanisms underlying their biological activity significantly depend on various parameters such as size, length, net charge, amino acid composition, hydrophobicity, amphiphilicity, secondary structures and oligomerization state [57,58]. On the other hand, the composition and features of the target membrane (lipids, cholesterol, charge, fluidity) also influence bioactivity by dictating the modalities of peptide interaction and the possible penetration and disruption of cell membranes [59,60]. Most ACPs, especially cationic and membrane-active ones, act through membrane disruption [2]. However, other ACPs, usually highly charged peptides, exhibit anticancer properties through the mediation of intracellular targets without necessarily inducing membrane disruption [2]. Although the mechanisms of action may differ for each ACP, this can be considered an advantage because it reduces the likelihood of developing resistance. Indeed, a recent work reviewed 21 AMPs with anticancer activity derived from fish, including peptide families like hepcidins, piscidins and pardaxin, as having high potential for targeting cancer cells [61]. To achieve this goal, AMPs must have the right combination of characteristics, including cytotoxicity against cancer cells without affecting normal healthy cells.

Accordingly, in this study, we explored the potential anticancer activity of the myristoylated (myr) lipopeptides Myr-A, Myr-B and Myr-C, along with their corresponding unmodified peptides Pep-A, Pep-B and Pep-C. Both myr and unmodified peptides were derived from the peptide KHS-Cnd, a mutant of the natural peptide chionodracine isolated from an Antarctic fish [34,37]. The choice of these lipopeptides was driven by our previous studies showing their ability to selectively interact with biological membrane-mimicking synthetic phospholipids, without being particularly cytotoxic to primary human fibroblasts or hemolytic against rabbit erythrocytes [40]. The length and saturation of the lipid moiety are key determinants of lipopeptide activity: optimal activity has typically been observed for saturated acyl chains, such as myristic acid (C14:0), which provide a balance between membrane affinity and biological selectivity [62]. Increasing the chain length has been reported to enhance hydrophobicity, which can lead to peptide aggregation, reduced selectivity and increased cytotoxicity. On the other hand, unlike saturated chains, which enhance peptide insertion and membrane destabilization, unsaturated ones, not adopting a linear conformation, reduce packing within the membrane lipid bilayer, thereby decreasing membrane-disruptive effects [63].

Notably, this study demonstrated that all myr peptides possessed a strong cytotoxic effect on HeLa cells at the highest tested doses (22.5 and 50 µM). Similar data on HeLa cells have been reported in the case of the cationic peptide LevHemB1, a novel AMP derived from the hemocyanin of *Litopenaeus vannamei*, which was cytotoxic at a concentration of 50 μM against the HeLa cell line, resulting in a cell viability percentage of approximately 30% [64]. The same effect was not observed in the normal liver cell line THLE-3, for which the viability percentage using the peptide at 50 μM did not drop below 70% [64].

Moreover, the exposure of HeLa cells to non-myr peptides did not result in any cytotoxic effect, even at the highest doses. Interestingly, adding myristic acid to the N-terminus of peptides caused them to acquire antitumoral activity, which was in line with a study conducted by Li and colleagues [42], demonstrating that the CM4 peptide (an AMP isolated from the hemolymph of the silkworm *Bombyx mori*), following myristoylation, had an increased anticancer activity against the breast cancer cell lines MCF-7, MDA-MB-231 and MX-1. Hence, myristoylation appeared to increase CM4 peptide anticancer activity through a stronger cell membrane interaction and by targeting mitochondria [42]. Unlike our non-myr peptides, which are short, highly flexible and only weakly active, the non-myr CM4 is a longer, well-structured peptide adopting a stable amphipathic α-helix and is intrinsically bioactive. This structural organization allows CM4 to interact with the cancer cell membrane through electrostatic interactions involving positively charged residues, although its ability to penetrate into cells has only been enhanced by myristoylation [42]. Conversely, for KHS-Cnd-derived peptides, the lipid moiety is essential for conferring biological activity, thus promoting membrane association, which appears to resemble the main mechanism of action. Consistent with this, previous molecular dynamics simulations in membrane models (POPC and POPC/POPG) have shown that Myr-B adopts an extended conformation parallel to the lipid surface, with its charged residues interacting with lipid phosphate groups. This membrane-associated conformation, which differs from that of Pep-B, provides the structural basis leading to the hypothesized stronger interaction with cancer cell membrane and the potential penetration properties of Myr-B [40].

As for the cancer cell specificity of lipopeptides, in line with the cytotoxic data of Bugli and colleagues [40], all myr peptides that we tested at the highest doses against primary human fibroblasts FB789 did not show the cytotoxicity observed for HeLa cells. Moreover, FB789 cells treated with non-myr peptides did not exhibit any significant cytotoxic effect, even at the highest tested dose, thus supporting the key role of N-myristoylation for the anticancer activity of peptides. To strengthen this data, all myr and unmodified peptides were also tested against another normal cell line, namely human dermal fibroblasts (HDFs). Notably, HDF cell viability did not show percentage values lower than 87% for any of the six studied peptides, thus confirming the selective action of myr peptides against HeLa cells. Among these lipopeptides, Myr-B was less hemolytic than Myr-C based on our previous data [40] and more cytotoxic than Myr-A against HeLa cells, as herein reported. Therefore, it was selected for further analyses aimed at evaluating the morphological/cellular and molecular signatures underlying the anticancer activity of Myr-B at the IC_50_ dose. Notably, this concentration was selected to study the biological processes and molecular pathways influenced by the peptide, thus avoiding the off-target effects and toxicity that emerge at the highest tested dose.

When exposed to HeLa cells at the IC_50_ dose, Myr-B impaired their cell membrane integrity, as documented by the elevated LDH release into the cell culture medium, and consequently induced necrosis, as shown by cell swelling, plasma membrane ruffling and rupture, causing the leakage of intracellular material. Interestingly, we observed that HeLa cells appeared more sensitive to the Myr-B peptide than Caco-2 cells, likely due to differences in membrane composition and organization between the two cell types. Notably, the lipopeptide did not induce necrosis in Caco-2 cells, as no evidence of cell membrane damage or leakage of intracellular contents was detected following treatment. These findings suggest that the biological effects of the Myr-B peptide are influenced by the cellular context and may depend on distinct modes of peptide–membrane interaction that elicit different cellular responses.

Unlike apoptosis, all the characteristics observed in Myr-B-treated HeLa cells resemble uncontrolled and non-programmed cell death, usually induced by harmful agents causing chemical and/or physical damage [2]. However, cell death by necrosis in HeLa cells is in line with the expected membranolytic activity of the Myr-B peptide, which has been previously reported to underlie its antifungal action [40] and which has been tested here, for the first time, against cancer cells. Moreover, this is also consistent with the literature data showing the regulation of tumor necrosis by ACPs exhibiting membrane-disruptive mechanisms, such as lactoferrin B-derived peptide, mellitin and cathelicidin [65,66,67]. However, previous studies have not always distinguished between necrosis and necroptosis or immune cell death, which generally occurs when apoptosis is inhibited and can trigger molecular signaling cascades with potential anticancer effects that are capable of killing treatment-resistant cells [68].

Consistently, morphological analysis alone does not define cell death, and ACPs are known to activate a series of underlying molecular mechanisms. To thoroughly investigate the effects induced by Myr-B at the IC_50_ dose in HeLa cells, we used high-throughput proteomics, thus contributing to an emerging field of research [69]. Although there are hundreds of studies describing the phenotypic effects of AMPs on cancer cells, the subset that has used unbiased and hypothesis-free proteomic approaches to determine the underlying molecular changes is currently very limited [70,71].

Taking advantage of shotgun LFQ proteomics combined with multiple statistical and bioinformatic analyses, distinct protein expression profiles were identified in association with each treatment condition: Myr-B- or Pep-B-treated cells. Searching for distinct proteomic signatures, multivariate analysis further revealed that Myr-B and the corresponding non-myr Pep-B modulate cellular pathways through distinct mechanisms. Bioinformatic enrichment showed functions perturbed by both peptide treatments but also some peptide-specific regulations. Pep-B mainly affected pathways involved in miRNA biogenesis, rRNA maturation and tRNA processing, with no evidence of cell death. Although the modulation of RNA networks has been widely explored in the context of anticancer strategies and innovative therapeutics [72,73,74,75], our data may suggest a general RNA metabolic adaptation in HeLa cells supported by Pep-B. In contrast, Myr-B treatment enriched a unique functional term, specifically the ASAP complex, which is composed of three core proteins: RNA-binding protein with serine-rich domain 1 (RNPS1), apoptotic chromatin condensation inducer 1 (ACIN1) and Sin3-associated protein of 18 kDa (SAP18). Together, these proteins form a functional module-interfacing post-transcriptional RNA regulator and programmed cell death pathway [76]. The ASAP complex is known to bind to RNA in a sequence-independent manner and is recruited to the exon junction complex (EJC), a messenger ribonucleoprotein complex involved in post-transcriptional regulation [77]. The three ASAP subunits (ACIN1, RNPS1 and SAP18) have been individually implicated in transcriptional regulation, pre-mRNA splicing and mRNA quality control [77]. Other studies suggest that the ASAP complex is involved in regulating both mRNA processing (splicing) and programmed cell death; in fact, it acts by inhibiting splicing mediated by other factors and accelerating cell death, disassembling after inducing apoptosis [76,78]. However, the mechanism of action for the ASAP complex remains to be fully understood, particularly whether its components play their regulatory role on RNA processing and apoptosis individually or as subunits of the complex. In our study, differential proteomics revealed that exposing HeLa cells to Myr-B resulted in the under-expression of all three core ASAP complex proteins (RNPS1, ACIN1, SAP18). This under-expression may reflect either a decreased stability of the complex under Myr-B treatment or an active downregulation of its components as part of the peptide’s mechanism of action, thereby tilting regulatory networks towards cell fate decisions that differ from those initiated by Pep-B. Interestingly, this finding resonates with the concept that perturbations in splicing-associated regulatory complexes can have broad consequences for both RNA processing and apoptotic susceptibility. Specifically, Michelle and colleagues reported increased levels of pro-apoptotic regulators such as Bcl-x, Bim and Mcl1 after the depletion of ASAP components through RNA interference [79]. Given that the deregulation of RNA processing and splicing machinery is increasingly recognized as a hallmark of cancer vulnerability and a fertile ground for therapeutic intervention [80,81,82], the ability of Myr-B to modulate this axis is especially intriguing. Proteomic analysis carried out here provides a valuable starting point for establishing a broader molecular framework for understanding the anticancer potential of the Myr-B peptide and formulating mechanistic hypotheses that can guide future investigations. In particular, the ASAP complex, as a reasonable intracellular target of Myr-B, warrants further investigation to determine whether the under-expression of its main proteins correlates with an impaired alternative splicing, which could consequently lead to a susceptibility to programmed cell death as a likely secondary stress response rather than as a direct mechanism. However, these observations should be considered preliminary, particularly in light of the apparent discrepancy between the proteomic findings and LDH/morphological results observed in HeLa cells. Therefore, any mechanistic interpretation based on the proteomic data should be considered exploratory and requires further functional validation to define the sequence of cell death pathways.

In conclusion, we demonstrated that the lipid modification of Pep-B profoundly reshapes the regulatory landscape engaged by the treatment, with a more specific effect on cancer cells in terms of the mechanism of Myr-B action. In particular, myristoylation helps the peptide to penetrate and modify/damage the membrane of HeLa cancer cells, to induce cell death predominantly by necrosis, as confirmed by our morphological/cellular analysis, and to cause an intracellular proteomic perturbation. This study indicated that combining the rational modular design of a natural AMP from a new biological source with peptide modification through N-myristoylation is a promising strategy for developing selective ACPs, making the Myr-B peptide an attractive anticancer drug for future investigations.

## 4. Materials and Methods

### 4.1. Peptides

The peptides were designed by Bugli et al. [40] and have the following sequences: Pep-A (WFGKLYRGITK), Pep-B (WRGITKVVKKV) and Pep-C (WVVKKVKGLLK). These three peptides were conjugated at the N-terminal with myristic acid (Myr), a saturated fatty acid with the chemical formula CH_3_(CH_2_)_12_COOH, yielding the three corresponding lipopeptides: Myr-A (Myr-WFGKLYRGITK), Myr-B (Myr-WRGITKVVKKV) and Myr-C (Myr-WVVKKVKGLLK). All peptides were synthesized with a purity greater than 98% and were provided by CASLO Aps (Kongens Lyngby, Denmark). Prior to use, each peptide was dissolved in sterile, filtered AccuGENE™ water (Lonza, Basel, Switzerland), and peptide concentrations were determined by measuring the absorbance at 280 nm using a Jasco V-630 spectrophotometer (Jasco International Co., Ltd., Tokyo, Japan).

### 4.2. Cell Cultures

The primary human FB789 fibroblast and primary human dermal fibroblasts (HDFs) were cultured at 37 °C with 5% CO_2_ in Dulbecco’s Modified Eagle Medium (DMEM) supplemented with 10% (*v*/*v*) Fetal Bovine Serum (FBS), 1% (*w*/*v*) glutamine and 1% (*w*/*v*) penicillin–streptomycin [83,84]. The human cervical cancer HeLa cell line (ATCC CCL-2) was cultured under the same conditions (37 °C, 5% CO_2_), but in Roswell Park Memorial Institute (RPMI) 1640 medium supplemented with 10% (*v*/*v*) FBS, 1% (*w*/*v*) glutamine, 1% (*w*/*v*) penicillin–streptomycin, and 0.2% (*w*/*v*) NaHCO_3_ [85]. The human colon cancer Caco-2 cell line (ATCC HTB-37) was cultured in DMEM supplemented with 10% (*v*/*v*) FBS, 1% (*w*/*v*) glutamine, 1% (*w*/*v*) penicillin–streptomycin, 1% Non-Essential Amino Acids and 1% sodium pyruvate and maintained at 37 °C in a humidified incubator with 5% CO_2_ [86].

### 4.3. Cytotoxicity Assay

Cells were seeded into a 96-well flat-bottom plate (at a density of 10^4^ cells per well in 100 μL of the medium) and incubated at 37 °C with 5% CO_2_ for 24 h. After this incubation, the medium was removed, and fresh medium containing the peptides at concentrations of 2.5, 7.5, 22.5 and 50 μM was added. The cells were then incubated at 37 °C with 5% CO_2_ for 3 h or 24 h. Following this, the medium was removed, and 100 μL of 3-(4,5-dimethylthiazol-2-yl)-2,5-diphenyltetrazolium bromide (MTT) solution (0.5 mg/mL) was added to each well for 3 h. The MTT solution was then removed, and 100 μL of dimethyl sulfoxide (DMSO) was added to dissolve the formazan crystals formed at the bottom of the wells. To ensure complete dissolution, the plate was incubated at 37 °C for 15 min. Absorbance was measured at 595 nm using an Epoch2 microplate spectrophotometer (BioTek, Winooski, VT, USA). Cell viability was expressed as the percentage of viable cells at each peptide concentration relative to untreated cells (negative control). The positive control consisted of cells grown in the absence of peptides but in the presence of 2% (*w*/*v*) NaN_3_. The cytotoxicity assay was performed on at least two biological replicates, each repeated in quintuplicate. Statistical analysis was performed using one-way ANOVA followed by Dunnett’s post hoc test (GraphPad Prism 11.0).

### 4.4. LDH Measurement

Cells were seeded on 96-well plates at a density of 10^4^ cells per well in 100 μL of the medium and, after overnight incubation, were treated with 38 μM Myr-B or Pep-B for 3 h. LDH release was determined using the CyQUANT™ LDH Cytotoxicity Assay (Invitrogen, Carlsbad, CA, USA), according to the manufacturer’s protocol. Absorbance was subsequently measured at 490 nm and 600 nm using an Epoch2 microplate reader (BioTek, Winooski, VT, USA). The LDH assay was performed on at least two biological replicates, each repeated in quintuplicate. Statistical analysis was performed using one-way ANOVA followed by Dunnett’s post hoc test (GraphPad Prism 11.0).

### 4.5. Scanning Electron Microscopy (SEM)

Cells were seeded on sterile glass coverslips inserted in 24-well cell culture plates at a density of 6 × 10^4^ cells per well in 100 μL of the medium. The cells were cultured at 37 °C with 5% CO_2_ for 24 h and treated with 38 µM Myr-B or Pep-B for 3 h. Negative control (untreated cells) was carried out by adding fresh medium, whereas positive control was obtained by treating cells with 2% (*w*/*v*) NaN_3_. Then, the cells were washed two times with PBS and fixed using 2.5% glutaraldehyde and 2% paraformaldehyde in 0.1 M cacodylate buffer, pH 7.2, at 4 °C for 3 h. After three washes with cacodylate buffer, the samples underwent dehydration by gradually increasing the concentration from 10% to 100% of ethanol solutions. After dehydration, the samples were dried using a Critical Point Dryer (CPD Emitech K850, Quorum Technologies Ltd., Kent, UK). Once dried, the samples were mounted on cylindrical aluminum stubs using conductive carbon adhesive tape, coated with gold using MED10 Sputtering (Balzers Union, Balzers, Liechtenstein) and then examined using a Jeol JSM 6010LA (Tokyo, Japan) at an accelerating voltage of 5 kV. Image acquisition and processing were performed using the inTouchScope Interface software.

### 4.6. Untargeted Proteomics and Bioinformatics Data Analysis

Proteomes were extracted from HeLa cells treated with the two peptides and the untreated control. Protein digestion was performed through on-filter proteolysis using S-Trap™ micro-spin columns (Protifi, Huntington, WV, USA) as published [87]. Peptide mixtures were analyzed through liquid chromatography–tandem mass spectrometry (LC–MS/MS) using an Orbitrap Exploris 240 coupled with a Vanquish Neo UHPLC system (both Thermo Fisher Scientific, Waltham, MA, USA). Instrument parameters for LC–MS/MS analysis followed the configuration reported in a previous publication [88]. Each condition consisted of n = 4 biological replicates, and each sample was analyzed by LC–MS/MS in technical duplicates. The resulting *.raw* files were processed using the label-free quantification (LFQ) approach within the MaxQuant (v2.7.0.0) platform. Protein identification was performed against the UniProt human proteome database (UP000005640; 20,402 canonical proteins, downloaded in February 2025) using the Andromeda search engine, applying a 1% false discovery rate (FDR) at both protein and peptide levels. Subsequent data normalization and elaboration procedures were carried out in Perseus (v2.1.3.0) [89]. Common contaminants, reverse hits and peptides identified solely by site modification were removed. Proteins were retained only if their identification was supported by at least one unique peptide. LFQ intensities were log2-transformed, and missing values were imputed using random numbers drawn from a Gaussian distribution (width = 0.3; downshift = 1.8). The protein matrix was exported and used for further statistical and bioinformatics analyses.

Partial Least Squares Discriminant Analysis (PLS-DA) was first performed to detect possible outliers using MetaboAnalyst 6.0. After the identification of one outlier, the biplot of PLS-DA and the relative loadings (top 20 features) were obtained to analyze the degree of separation among groups and the features explaining the obtained variance [90,91]. Furthermore, the performance of the PLS-DA model was estimated through the 5-fold cross-validation method by calculating the fitness (R2), predictive ability (Q2) and accuracy of the model [92]. Heatmaps and hierarchical clustering of the top-selected features were generated using the ClustVis web tool, applying unit variance scaling to rows and correlation distance and average linkage to both rows and columns [93]. Differential expression between pairwise conditions was assessed using GraphPad Prism 11.0 through multiple unpaired *t*-tests with Welch correction. Proteins were considered significantly varied using a nominal *p*-value threshold of 0.01 (−log10 *p*-value > 2), combined with a difference cutoff < −0.2 (downregulated) or >0.2 (upregulated). The use of nominal *p*-values rather than multiple-testing-adjusted values was motivated by the exploratory nature of this study and by the limited sample size. In such contexts, stringent multiple-testing corrections can be excessively conservative and may fail to detect true positives, potentially masking biologically relevant but moderate protein abundance changes [94]. The features identified in this work may therefore be useful and can be explored in future studies. Metascape was used to generate protein–protein interaction (PPI) networks, and gene ontology (GO) enrichment analysis was applied to the network to extract biological meanings from the network component, where the best *p*-value for non-redundant terms was retained. Overlaps between gene lists were visualized using circos plots [95]. Pearson correlation analysis was performed using SRplot through correlation of the difference value lists of Myr-B vs. CTRL and Pep-B vs. CTRL comparisons.

Multiple comparison analysis was performed with GraphPad Prism 11.0 using two-way ANOVA with Tukey correction to statistically compare and validate abundance differences in the ASAP complex members in Myr-B samples versus Pep-B and CTRL samples. Adjusted *p*-values were considered significant if at least lower than 0.05.

## Figures and Tables

**Table 1:** N-myristoylated Myr-A, Myr-B and Myr-C peptide IC_50_ values for human cervical cancer HeLa cells after 3 h and 24 h of treatment.

IC_50_ (µM Mean ± SD)		
Peptide	3 h	24 h
Myr-A	45.73 ± 13.73	47.36 ± 0.75
Myr-B	38.50 ± 3.85	41.72 ± 11.34
Myr-C	33.68 ± 3.46	32.12 ± 0.30

## Data Availability

The mass spectrometry proteomics data are available through the PRIDE [96] partner repository with the dataset identifier PXD074461.

## Supplementary Materials

The following supporting information can be downloaded at https://www.mdpi.com/article/10.3390/ijms27093918/s1.

## References

[1] Yang R., Ma X., Peng F., Wen J., Allahou L.W., Williams G.R., Knowles J.C., Poma A. (2025). Advances in Antimicrobial Peptides: From Mechanistic Insights to Chemical Modifications. Biotechnol. Adv..

[2] Qu B., Yuan J., Liu X., Zhang S., Ma X., Lu L. (2024). Anticancer Activities of Natural Antimicrobial Peptides from Animals. Front. Microbiol..

[3] Shabir U., Ali S., Magray A.R., Ganai B.A., Firdous P., Hassan T., Nazir R. (2018). Fish Antimicrobial Peptides (AMP’s) as Essential and Promising Molecular Therapeutic Agents: A Review. Microb. Pathog..

[4] Lucchetti D., Rinaldi R., Artemi G., Salvia R., De Stefano F., Scieuzo C., Falabella P., Sgambato A. (2025). Peptide Fractions Extracted from the Hemolymph of *Hermetia illucens* Inhibit Growth and Motility and Enhance the Effects of Traditional Chemotherapeutics in Human Colorectal Cancer Cells. Int. J. Mol. Sci..

[5] Varela-Quitián Y.F., Mendez-Rivera F.E., Bernal-Estévez D.A. (2025). Cationic Antimicrobial Peptides: Potential Templates for Anticancer Agents. Front. Med..

[6] Verma R., Yadav S., Likhita J., Kumar S., Shelke M., Pal R., Prajapati M.K., Behera P.R., Singh Y., Puyam A. (2026). Deciphering the Molecular Roles of Antimicrobial Peptides in Plant Innate Immunity and Their Potential Applications in Crop Protection. Plant Physiol. Biochem..

[7] Zhang Q. (2025). Antimicrobial Peptides: From Discovery to Developmental Applications. Appl. Environ. Microbiol..

[8] Kim S.H., Min Y.-H., Park M.C. (2025). Antimicrobial Peptides: Current Status, Mechanisms of Action, and Strategies to Overcome Therapeutic Limitations. Microorganisms.

[9] Huang X., Li G. (2023). Antimicrobial Peptides and Cell-Penetrating Peptides: Non-Antibiotic Membrane-Targeting Strategies Against Bacterial Infections. Infect. Drug Resist..

[10] Gagandeep K.R., Balenahalli Narasingappa R., Vishnu Vyas G. (2024). Unveiling Mechanisms of Antimicrobial Peptide: Actions beyond the Membranes Disruption. Heliyon.

[11] Ma X., Wang Q., Ren K., Xu T., Zhang Z., Xu M., Rao Z., Zhang X. (2024). A Review of Antimicrobial Peptides: Structure, Mechanism of Action, and Molecular Optimization Strategies. Fermentation.

[12] Rima M., Rima M., Fajloun Z., Sabatier J.-M., Bechinger B., Naas T. (2021). Antimicrobial Peptides: A Potent Alternative to Antibiotics. Antibiotics.

[13] Roque-Borda C.A., Zhang Q., Nguyen T.P.T., Nguyen T.T.H., Medhi H., Rodrigues H.L.d.S., Canales Carnero C.S., Sutherland D., Helmy N.M., Araveti P.B. (2026). Synergistic Combinations of Antimicrobial Peptides and Conventional Antibiotics: A Strategy to Delay Resistance Emergence in World Health Organization Priority Bacteria. Pharmacol. Rev..

[14] Brown J.S., Amend S.R., Austin R.H., Gatenby R.A., Hammarlund E.U., Pienta K.J. (2023). Updating the Definition of Cancer. Mol. Cancer Res..

[15] Anand U., Dey A., Chandel A.K.S., Sanyal R., Mishra A., Pandey D.K., De Falco V., Upadhyay A., Kandimalla R., Chaudhary A. (2023). Cancer Chemotherapy and beyond: Current Status, Drug Candidates, Associated Risks and Progress in Targeted Therapeutics. Genes Dis..

[16] Kannan K., Srinivasan A., Kannan A., Ali N. (2025). The Underlying Mechanisms and Emerging Strategies to Overcome Resistance in Breast Cancer. Cancers.

[17] Tufail M., Hu J.-J., Liang J., He C.-Y., Wan W.-D., Huang Y.-Q., Jiang C.-H., Wu H., Li N. (2024). Hallmarks of Cancer Resistance. iScience.

[18] Hanahan D. (2026). Hallmarks of Cancer—Then and Now, and Beyond. Cell.

[19] Vasan N., Baselga J., Hyman D.M. (2019). A View on Drug Resistance in Cancer. Nature.

[20] Li J., Hu J., Yang Y., Zhang H., Liu Y., Fang Y., Qu L., Lin A., Luo P., Jiang A. (2025). Drug Resistance in Cancer: Molecular Mechanisms and Emerging Treatment Strategies. Mol. Biomed..

[21] Zare-Zardini H., Saberian E., Jenča A., Ghanipour-Meybodi R., Jenča A., Petrášová A., Jenčová J. (2024). From Defense to Offense: Antimicrobial Peptides as Promising Therapeutics for Cancer. Front. Oncol..

[22] Dong Z., Zhang X., Zhang Q., Tangthianchaichana J., Guo M., Du S., Lu Y. (2024). Anticancer Mechanisms and Potential Anticancer Applications of Antimicrobial Peptides and Their Nano Agents. Int. J. Nanomed..

[23] Tornesello A.L., Borrelli A., Buonaguro L., Buonaguro F.M., Tornesello M.L. (2020). Antimicrobial Peptides as Anticancer Agents: Functional Properties and Biological Activities. Molecules.

[24] Parchebafi A., Tamanaee F., Ehteram H., Ahmad E., Nikzad H., Haddad Kashani H. (2022). The Dual Interaction of Antimicrobial Peptides on Bacteria and Cancer Cells; Mechanism of Action and Therapeutic Strategies of Nanostructures. Microb. Cell Fact..

[25] Arias M., Haney E.F., Hilchie A.L., Corcoran J.A., Hyndman M.E., Hancock R.E.W., Vogel H.J. (2020). Selective Anticancer Activity of Synthetic Peptides Derived from the Host Defence Peptide Tritrpticin. Biochim. Biophys. Acta (BBA)-Biomembr..

[26] Trinidad-Calderón P.A., Varela-Chinchilla C.D., García-Lara S. (2021). Natural Peptides Inducing Cancer Cell Death: Mechanisms and Properties of Specific Candidates for Cancer Therapeutics. Molecules.

[27] Ghaly G., Tallima H., Dabbish E., Badr ElDin N., Abd El-Rahman M.K., Ibrahim M.A.A., Shoeib T. (2023). Anti-Cancer Peptides: Status and Future Prospects. Molecules.

[28] Lin L., Chi J., Yan Y., Luo R., Feng X., Zheng Y., Xian D., Li X., Quan G., Liu D. (2021). Membrane-Disruptive Peptides/Peptidomimetics-Based Therapeutics: Promising Systems to Combat Bacteria and Cancer in the Drug-Resistant Era. Acta Pharm. Sin. B.

[29] Rinaldi R., Laurino S., Salvia R., Russi S., De Stefano F., Galasso R., Sgambato A., Scieuzo C., Falco G., Falabella P. (2025). Biological Activity of Peptide Fraction Derived from *Hermetia illucens* L. (Diptera: Stratiomyidae) Larvae Haemolymph on Gastric Cancer Cells. Int. J. Mol. Sci..

[30] Yu H.-H., Wu L.-Y., Hsu P.-L., Lee C.-W., Su B.-C. (2024). Marine Antimicrobial Peptide Epinecidin-1 Inhibits Proliferation Induced by Lipoteichoic Acid and Causes Cell Death in Non-Small Cell Lung Cancer Cells via Mitochondria Damage. Probiotics Antimicrob. Proteins.

[31] Chen Y.-P., Shih P.-C., Feng C.-W., Wu C.-C., Tsui K.-H., Lin Y.-H., Kuo H.-M., Wen Z.-H. (2021). Pardaxin Activates Excessive Mitophagy and Mitochondria-Mediated Apoptosis in Human Ovarian Cancer by Inducing Reactive Oxygen Species. Antioxidants.

[32] Hilchie A.L., Hoskin D.W., Power Coombs M.R., Matsuzaki K. (2019). Anticancer Activities of Natural and Synthetic Peptides. Antimicrobial Peptides.

[33] Kotalík K., Etrych T. (2026). KLAK Peptide in Anticancer Therapy: Achieving Cancer Cell Apoptosis via Mitochondrial Membrane Disruption Using Homing Domains Introduction and Other Modifications. Eur. J. Med. Chem..

[34] Buonocore F., Randelli E., Casani D., Picchietti S., Belardinelli M.C., de Pascale D., De Santi C., Scapigliati G. (2012). A Piscidin-like Antimicrobial Peptide from the Icefish *Chionodraco hamatus* (Perciformes: Channichthyidae): Molecular Characterization, Localization and Bactericidal Activity. Fish Shellfish Immunol..

[35] Della Pelle G., Perà G., Belardinelli M.C., Gerdol M., Felli M., Crognale S., Scapigliati G., Ceccacci F., Buonocore F., Porcelli F. (2020). Trematocine, a Novel Antimicrobial Peptide from the Antarctic Fish *Trematomus bernacchii*: Identification and Biological Activity. Antibiotics.

[36] Shin S.C., Ahn I.H., Ahn D.H., Lee Y.M., Lee J., Lee J.H., Kim H.-W., Park H. (2017). Characterization of Two Antimicrobial Peptides from Antarctic Fishes (*Notothenia coriiceps* and *Parachaenichthys charcoti*). PLoS ONE.

[37] Olivieri C., Buonocore F., Picchietti S., Taddei A.R., Bernini C., Scapigliati G., Dicke A.A., Vostrikov V.V., Veglia G., Porcelli F. (2015). Structure and Membrane Interactions of Chionodracine, a Piscidin-like Antimicrobial Peptide from the Icefish *Chionodraco hamatus*. Biochim. Biophys. Acta (BBA)-Biomembr..

[38] Olivieri C., Bugli F., Menchinelli G., Veglia G., Buonocore F., Scapigliati G., Stocchi V., Ceccacci F., Papi M., Sanguinetti M. (2018). Design and Characterization of Chionodracine-Derived Antimicrobial Peptides with Enhanced Activity against Drug-Resistant Human Pathogens. RSC Adv..

[39] Buonocore F., Picchietti S., Porcelli F., Della Pelle G., Olivieri C., Poerio E., Bugli F., Menchinelli G., Sanguinetti M., Bresciani A. (2019). Fish-Derived Antimicrobial Peptides: Activity of a Chionodracine Mutant against Bacterial Models and Human Bacterial Pathogens. Dev. Comp. Immunol..

[40] Bugli F., Massaro F., Buonocore F., Saraceni P.R., Borocci S., Ceccacci F., Bombelli C., Di Vito M., Marchitiello R., Mariotti M. (2022). Design and Characterization of Myristoylated and Non-Myristoylated Peptides Effective against *Candida* spp. Clinical Isolates. Int. J. Mol. Sci..

[41] Rounds T., Straus S.K. (2020). Lipidation of Antimicrobial Peptides as a Design Strategy for Future Alternatives to Antibiotics. Int. J. Mol. Sci..

[42] Li C., Liu H., Yang Y., Xu X., Lv T., Zhang H., Liu K., Zhang S., Chen Y. (2018). N-Myristoylation of Antimicrobial Peptide CM4 Enhances Its Anticancer Activity by Interacting with Cell Membrane and Targeting Mitochondria in Breast Cancer Cells. Front. Pharmacol..

[43] Li N., Jiang X., Ma X., Qiu X., Chang H., Qiao Y., Luo H., Zhang Q. (2023). Antimicrobial Peptides CS-Piscidin-Induced Cell Death Involves Activation of RIPK1/PARP, and Modification with Myristic Acid Enhances Its Stability and Tumor-Targeting Capability. Discov. Oncol..

[44] Liu Y., Li S., Shen T., Chen L., Zhou J., Shi S., Wang Y., Zhao Z., Liao C., Wang C. (2020). N-Terminal Myristoylation Enhanced the Antimicrobial Activity of Antimicrobial Peptide PMAP-36PW. Front. Cell. Infect. Microbiol..

[45] Ji D., Kamalden T.A., del Olmo-Aguado S., Osborne N.N. (2011). Light- and Sodium Azide-Induced Death of RGC-5 Cells in Culture Occurs via Different Mechanisms. Apoptosis.

[46] Zuo Y., Hu J., Xu X., Gao X., Wang Y., Zhu S. (2019). Sodium Azide Induces Mitochondria-mediated Apoptosis in PC12 Cells through Pgc-1α-associated Signaling Pathway. Mol. Med. Rep..

[47] Weyermann J., Lochmann D., Zimmer A. (2005). A Practical Note on the Use of Cytotoxicity Assays. Int. J. Pharm..

[48] Chan F.K.-M., Moriwaki K., De Rosa M.J., Snow A., Lenardo M. (2013). Detection of Necrosis by Release of Lactate Dehydrogenase Activity. Immune Homeostasis.

[49] Abdullah K.M., Sharma G., Singh A.P., Siddiqui J.A. (2025). Nanomedicine in Cancer Therapeutics: Current Perspectives from Bench to Bedside. Mol. Cancer.

[50] Mantri S., Doshi G.M. (2025). Reactivating Apoptotic Pathways in Cancer: A Review of Novel Therapeutic Approaches. Eur. J. Pharmacol..

[51] Neophytou C.M., Trougakos I.P., Erin N., Papageorgis P. (2021). Apoptosis Deregulation and the Development of Cancer Multi-Drug Resistance. Cancers.

[52] Lei Z., Tian Q., Teng Q., Wurpel J.N.D., Zeng L., Pan Y., Chen Z. (2023). Understanding and Targeting Resistance Mechanisms in Cancer. MedComm.

[53] Liu B., Zhou H., Tan L., Siu K.T.H., Guan X.-Y. (2024). Exploring Treatment Options in Cancer: Tumor Treatment Strategies. Signal Transduct. Target. Ther..

[54] Gaspar D., Veiga A.S., Castanho M.A.R.B. (2013). From Antimicrobial to Anticancer Peptides. A Review. Front. Microbiol..

[55] Wang M., Xia H., Wang C., Zhang T., Zhang M., Li X., Peng C., Jing T., Wang Y., Peng L. (2025). From Precision Synthesis to Cross-Industry Applications: The Future of Emerging Peptide Technologies. Pharmacol. Res..

[56] Li Q., Chao W., Qiu L. (2025). Therapeutic Peptides: Chemical Strategies Fortify Peptides for Enhanced Disease Treatment Efficacy. Amino Acids.

[57] Gagat P., Ostrówka M., Duda-Madej A., Mackiewicz P. (2024). Enhancing Antimicrobial Peptide Activity through Modifications of Charge, Hydrophobicity, and Structure. Int. J. Mol. Sci..

[58] Mwangi J., Kamau P., Thuku R., Lai R. (2023). Design Methods for Antimicrobial Peptides with Improved Performance. Zool. Res..

[59] Avci F.G., Sariyar Akbulut B., Ozkirimli E. (2018). Membrane Active Peptides and Their Biophysical Characterization. Biomolecules.

[60] Giraldo-Lorza J.M., Leidy C., Manrique-Moreno M. (2024). The Influence of Cholesterol on Membrane Targeted Bioactive Peptides: Modulating Peptide Activity Through Changes in Bilayer Biophysical Properties. Membranes.

[61] Najm A.A., Azfaralarriff A., Eziwar Dyari H.R., Syed Alwi S.S., Khalili N., Othman B.A., Law D., Shahid M., Fazry S. (2022). A Systematic Review of Antimicrobial Peptides from Fish with Anticancer Properties. Pertanika J. Sci. Technol..

[62] Malina A., Shai Y. (2005). Conjugation of Fatty Acids with Different Lengths Modulates the Antibacterial and Antifungal Activity of a Cationic Biologically Inactive Peptide. Biochem. J..

[63] Helmy N.M., Davani-Davari D., Parang K. (2026). Fatty Acid-Conjugated Antimicrobial Peptides: Advances in Design, Activity, and Therapeutic Potential. J. Med. Chem..

[64] Liu S., Aweya J.J., Zheng L., Zheng Z., Huang H., Wang F., Yao D., Ou T., Zhang Y. (2022). LvHemB1, a Novel Cationic Antimicrobial Peptide Derived from the Hemocyanin of *Litopenaeus vannamei*, Induces Cancer Cell Death by Targeting Mitochondrial Voltage-Dependent Anion Channel 1. Cell Biol. Toxicol..

[65] Mahmoud M.M., Alenezi M., Al-Hejin A.M., Abujamel T.S., Aljoud F., Noorwali A., Awad I.A., Alkhaled M., Yacoub H.A. (2022). Anticancer Activity of Chicken Cathelicidin Peptides against Different Types of Cancer. Mol. Biol. Rep..

[66] Daniluk K., Kutwin M., Grodzik M., Wierzbicki M., Strojny B., Szczepaniak J., Bałaban J., Sosnowska M., Chwalibog A., Sawosz E. (2019). Use of Selected Carbon Nanoparticles as Melittin Carriers for MCF-7 and MDA-MB-231 Human Breast Cancer Cells. Materials.

[67] Zhou H., Forveille S., Sauvat A., Yamazaki T., Senovilla L., Ma Y., Liu P., Yang H., Bezu L., Müller K. (2016). The Oncolytic Peptide LTX-315 Triggers Immunogenic Cell Death. Cell Death Dis..

[68] Ye K., Chen Z., Xu Y. (2023). The Double-Edged Functions of Necroptosis. Cell Death Dis..

[69] Liscano Y., Oñate-Garzón J., Delgado J.P. (2020). Peptides with Dual Antimicrobial–Anticancer Activity: Strategies to Overcome Peptide Limitations and Rational Design of Anticancer Peptides. Molecules.

[70] Yang L.-B., Guo G., Tian Z.-Q., Zhou L.-X., Zhu L.-J., Peng J., Sun C.-Q., Huang M.-J. (2022). TMT-Based Quantitative Proteomic Analysis of the Effects of Novel Antimicrobial Peptide AMP-17 against *Candida albicans*. J. Proteom..

[71] Czaplewska P., Bogucka A., Macur K., Rybicka M., Rychłowski M., Fiołka M.J. (2023). Proteomic Response of A549 Lung Cancer Cell Line to Protein-Polysaccharide Complex Venetin-1 Isolated from Earthworm Coelomic Fluid. Front. Mol. Biosci..

[72] Romero-Cordoba S.L., Salido-Guadarrama I., Rodriguez-Dorantes M., Hidalgo-Miranda A. (2014). MiRNA Biogenesis: Biological Impact in the Development of Cancer. Cancer Biol. Ther..

[73] Costanzo M., Roviello G.N. (2025). Precision Therapeutics Through Bioactive Compounds: Metabolic Reprogramming, Omics Integration, and Drug Repurposing Strategies. Int. J. Mol. Sci..

[74] Sharifi H., Jafari Najaf Abadi M.H., Razi E., Mousavi N., Morovati H., Sarvizadeh M., Taghizadeh M. (2019). MicroRNAs and Response to Therapy in Leukemia. J. Cell. Biochem..

[75] Son S.W., Lee H.Y., Moeng S., Kuh H.J., Choi S.Y., Park J.K. (2020). Participation of MicroRNAs in the Treatment of Cancer with Phytochemicals. Molecules.

[76] Deka B., Singh K.K. (2017). Multifaceted Regulation of Gene Expression by the Apoptosis- and Splicing-Associated Protein Complex and Its Components. Int. J. Biol. Sci..

[77] Murachelli A.G., Ebert J., Basquin C., Le Hir H., Conti E. (2012). The Structure of the ASAP Core Complex Reveals the Existence of a Pinin-Containing PSAP Complex. Nat. Struct. Mol. Biol..

[78] Schwerk C., Prasad J., Degenhardt K., Erdjument-Bromage H., White E., Tempst P., Kidd V.J., Manley J.L., Lahti J.M., Reinberg D. (2003). ASAP, a Novel Protein Complex Involved in RNA Processing and Apoptosis. Mol. Cell. Biol..

[79] Michelle L., Cloutier A., Toutant J., Shkreta L., Thibault P., Durand M., Garneau D., Gendron D., Lapointe E., Couture S. (2012). Proteins Associated with the Exon Junction Complex Also Control the Alternative Splicing of Apoptotic Regulators. Mol. Cell. Biol..

[80] Bradley R.K., Anczuków O. (2023). RNA Splicing Dysregulation and the Hallmarks of Cancer. Nat. Rev. Cancer.

[81] Bashari A., Siegfried Z., Karni R. (2023). Targeting Splicing Factors for Cancer Therapy. RNA.

[82] Naro C., Ruta V., Sette C. (2025). Splicing Dysregulation: Hallmark and Therapeutic Opportunity in Pancreatic Cancer. Trends Mol. Med..

[83] Imperlini E., Celia C., Cevenini A., Mandola A., Raia M., Fresta M., Orrù S., Di Marzio L., Salvatore F. (2021). Nano-Bio Interface between Human Plasma and Niosomes with Different Formulations Indicates Protein Corona Patterns for Nanoparticle Cell Targeting and Uptake. Nanoscale.

[84] Imperlini E., Massaro F., Grifoni A., Maiurano F., Taddei A.R., Borocci S., Buonocore F., Porcelli F. (2024). Membrane Alteration, Anti-Virulence Properties and Metabolomic Perturbation of a Chionodracine-Derived Antimicrobial Peptide, KHS-Cnd, on Two Bacteria Models. Peptides.

[85] Roviello G.N., Roviello G., Musumeci D., Capasso D., Di Gaetano S., Costanzo M., Pedone C. (2014). Synthesis and Supramolecular Assembly of 1,3-Bis(1′-Uracilyl)-2-Propanone. RSC Adv..

[86] Imperlini E., Colavita I., Caterino M., Mirabelli P., Pagnozzi D., Del Vecchio L., Di Noto R., Ruoppolo M., Orrù S. (2013). The Secretome Signature of Colon Cancer Cell Lines. J. Cell. Biochem..

[87] Ruiz-Blázquez P., Fernández-Fernández M., Pistorio V., Martinez-Sanchez C., Costanzo M., Iruzubieta P., Zhuravleva E., Cacho-Pujol J., Ariño S., Del Castillo-Cruz A. (2024). Cathepsin D Is Essential for the Degradomic Shift of Macrophages Required to Resolve Liver Fibrosis. Mol. Metab..

[88] Costanzo M., Cevenini A., Kollipara L., Caterino M., Bianco S., Pirozzi F., Scerra G., D’Agostino M., Pavone L.M., Sickmann A. (2024). Methylmalonic Acidemia Triggers Lysosomal-Autophagy Dysfunctions. Cell Biosci..

[89] Costanzo M., Caterino M., Salvatori I., Manganelli V., Ferri A., Misasi R., Ruoppolo M. (2022). Proteome Data of Neuroblastoma Cells Overexpressing Neuroglobin. Data Brief.

[90] Costanzo M., Caterino M. (2023). Targeted Lipidomics Data of COVID-19 Patients. Data Brief.

[91] Pang Z., Lu Y., Zhou G., Hui F., Xu L., Viau C., Spigelman A.F., MacDonald P.E., Wishart D.S., Li S. (2024). MetaboAnalyst 6.0: Towards a Unified Platform for Metabolomics Data Processing, Analysis and Interpretation. Nucleic Acids Res..

[92] Costanzo M., Miceli M., Campesi I., Bianco S., Mazzarelli L.L., Migliorini S., Sarno L., Malesci R., Salomè S., Raimondi F. (2026). Metabolomics Highlights Reduced Glutamate and Acetylcarnitine in the Amniotic Fluid of Cytomegalovirus-Infected Pregnant Women. J. Proteome Res..

[93] Metsalu T., Vilo J. (2015). ClustVis: A Web Tool for Visualizing Clustering of Multivariate Data Using Principal Component Analysis and Heatmap. Nucleic Acids Res..

[94] Pascovici D., Handler D.C.L., Wu J.X., Haynes P.A. (2016). Multiple Testing Corrections in Quantitative Proteomics: A Useful but Blunt Tool. Proteomics.

[95] Zhou Y., Zhou B., Pache L., Chang M., Khodabakhshi A.H., Tanaseichuk O., Benner C., Chanda S.K. (2019). Metascape Provides a Biologist-Oriented Resource for the Analysis of Systems-Level Datasets. Nat. Commun..

[96] Perez-Riverol Y., Bandla C., Kundu D.J., Kamatchinathan S., Bai J., Hewapathirana S., John N.S., Prakash A., Walzer M., Wang S. (2025). The PRIDE Database at 20 Years: 2025 Update. Nucleic Acids Res..

